# Lipid Droplets, the Central Hub Integrating Cell Metabolism and the Immune System

**DOI:** 10.3389/fphys.2021.746749

**Published:** 2021-12-03

**Authors:** Wei Zhang, Linyong Xu, Ling Zhu, Yifan Liu, Siwei Yang, Mingyi Zhao

**Affiliations:** ^1^Department of Pediatrics, Third Xiangya Hospital, Central South University, Changsha, China; ^2^Xiangya Hospital, Central South University, Changsha, China; ^3^School of Life Sciences, Central South University, Changsha, China; ^4^Xiangya School of Medicine, Central South University, Changsha, China

**Keywords:** lipid droplets, immune cells, atherosclerosis, metabolism, immunity

## Abstract

Lipid droplets (LDs) are commonly found in various biological cells and are organelles related to cell metabolism. LDs, the number and size of which are heterogeneous across cell type, are primarily composed of polar lipids and proteins on the surface with neutral lipids in the core. Neutral lipids stored in LDs can be degraded by lipolysis and lipophagocytosis, which are regulated by various proteins. The process of LD formation can be summarized in four steps. In addition to energy production, LDs play an extremely pivotal role in a variety of physiological and pathological processes, such as endoplasmic reticulum stress, lipid toxicity, storage of fat-soluble vitamins, regulation of oxidative stress, and reprogramming of cell metabolism. Interestingly, LDs, the hub of integration between metabolism and the immune system, are involved in antitumor immunity, anti-infective immunity (viruses, bacteria, parasites, etc.) and some metabolic immune diseases. Herein, we summarize the role of LDs in several major immune cells as elucidated in recent years, including T cells, dendritic cells, macrophages, mast cells, and neutrophils. Additionally, we analyze the role of the interaction between LDs and immune cells in two typical metabolic immune diseases: atherosclerosis and Mycobacterium tuberculosis infection.

## Introduction

### Lipid Droplets

Lipid droplets (LDs), organelles related to cell metabolism that store lipids in different cells, vary greatly in number, size, and structure. LDs consist of the core of the neutral lipid surrounded by a polar lipid monolayer with the polar head group of phospholipids facing the cytosol their and acyl chains contacting the hydrophobic neutral lipid nucleus, while the surface of the LDs contains many different proteins that are related to the specific function of the LDs (Kory et al., 2016). Commonly found in most eukaryotic cells and some prokaryotic cells, even in the nuclei of some cells, LDs can store neutral lipids that can be degraded by lipolysis mediated by LDs-associated lipases or lipophagy mediated by molecular chaperones that recognize proteins on the surface of LDs and transfer them to lysosomes for degradation (Murphy, 2012). Not merely limited to energy storage, LDs play an important role in many physiological and pathological processes within the body, such as preventing the accumulation of toxic substances in the endoplasmic reticulum through regulation of the oxidative stress process, combining with mitochondria to participate in metabolic regulation, mediating cellular dysfunction and even modulating serious diseases caused by mutations in certain characteristic LD-related proteins (Welte and Gould, 2017).

**FIGURE 1:**
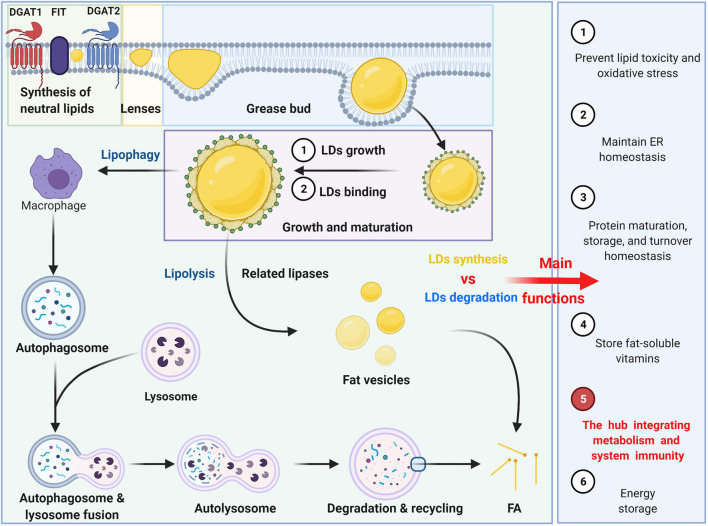
The formation, degradation and main functions of LDs. The formation can be summarized into several conceptual steps: synthesis of neutral lipids and formation of lenses, grease bud, growth and maturation of LDs. The degradation of LDs can be divided into two ways: lipophagy and lipolysis.

Currently, the process of lipid droplet formation (Figure 1), which follows the process of transferring from the formation of the oil phase to the cytoplasm of the water phase, is not very clear, but it can be summarized into several conceptual steps: synthesis of neutral lipids and formation of lenses, grease bud, growth and maturation of LDs (Walther et al., 2017; Olzmann and Carvalho, 2019). Neutral lipid synthesis induced by Nem1p-Spo7p plays a very important role in maintaining the lipid balance of the ER membrane, particularly by preventing excessive accumulation of lipids (Karanasios et al., 2013). The FIT protein may affect germination and promote germination from ER-appropriate LDs. There is also evidence that nascent LDs form a lens-like structure in the ER membrane, and in the absence of FIT proteins, nascent LDs cannot germinate from the ER but grow and remain in the membrane (Choudhary et al., 2015). There may be multiple proteins involved in the budding process. For example, PERILIPIN (PLIN)-3 is considered to be the primary regulator of LD formation (Skinner et al., 2009). Studies have shown that PLD1 and ERK2 are essential for the formation of LDs and enhancing insulin stimulation (Andersson et al., 2006). Previous studies indicated that TIP47, with its lipoid properties that functions similar to recombinant liposomes, plays a role in the development of LD biogenesis because inhibition of TIP47 blocks the maturation of LDs and reduces the incorporation of trimethylphenyl glycerol (Bulankina et al., 2009). LDs grow and expand by acquiring specific proteins. Large LDs are formed by at least two general mechanisms: the process of LD growth or LD binding to form a single and larger LD. The LD expansion pathway is triggered by the activation of ARF1/COP-I proteins on the LD surface, forming nano LDs and removing primarily phospholipids from the LD surface, increasing the surface tension and enabling the modified LDs to interact with the endoplasmic reticulum. This process then allows the formation of ER-LD membrane bridges as well as the migration of TG synthase to the surface for LD expansion (Wilfling et al., 2014). LDs can also fuse to achieve amplification, including diffusion mediated by Ostwald maturation. The transcription factor MLX is one of the targets localized to LDs. By binding to LDs, MLX coordinates lipid storage by regulating the expression of metabolic genes (Mejhert et al., 2020).

Neutral lipids stored in LDs are primarily degraded by two pathways: lipophagy and lipolysis. LD degradation can selectively target LDs through autophagy-mediated lipolysis (macrophages) by hydrolyzing triglycerides (TGs) into free fatty acids (FFAs) as an energy source (Olzmann and Carvalho, 2019). The LD-related proteins Perilipin 2 (PLIN2) and Perilipin 3 (PLIN3) are autophagic substrates that undergo autophagy degradation before lipolysis (Kaushik and Cuervo, 2015). It was found that a decrease in autophagy promotes lipid accumulation and further inhibits autophagy, increasing lipid retention (Robichaud et al., 2021). Lysosomes do not directly fuse with LDs but with autophagosomes containing LDs (Singh, 2010). In addition, LDs can be decomposed into multiple fat vesicles by related lipases. Phosphatidylcholine prevents lipid droplet polymerization, and the rate-limiting enzyme in phosphatidylcholine synthesis is activated by binding to LDs.

Oxysterols, the hydroxylated metabolites of cholesterol, were first identified as intermediates in bile acid metabolism, but their pleiotropic roles in immunity and inflammation draw more attention (Spann and Glass, 2013; Cyster et al., 2014; Aguilar-Ballester et al., 2020). The oxysterols mainly include 25-hydroxycholesterol, 7α,25-dihydroxycholesterol, 27-hydroxycholesterol and 7α,27-dihydroxycholesterol (Willinger, 2019). The oxysterols can involve the regulation of the LDs in immune cells. The crosstalk between the 25-hydroxycholesterol and RORα can regulate the LDs homeostasis in macrophages (Tuong et al., 2016).

In general, LDs, organelles that store neutral lipids, play an important role in energy metabolism, preventing lipid toxicity and oxidative stress (Young et al., 2013; Bosma et al., 2014). LDs can also maintain endoplasmic reticulum homeostasis by storing excess lipids, regulating the metabolism and homeostasis of their lipids or protein cargo and playing a role in protein maturation, storage, and turnover. Additionally, LDs can also provide corresponding physiological sites to store fat-soluble vitamins, but for other vitamins, this process is not clear (Welte and Gould, 2017). More importantly, LDs act as bioactive lipids that regulate inflammation and immunity and are the hub that integrates metabolism and systemic immunity. Therefore, herein, we summarize the important role of LDs in multiple immune cells and analyze the role of the interaction between LDs and immunity in several related diseases.

### Immunometabolism

The immune system provides defense, surveillance, and clearance functions for the body to prevent disease progression. While conducting these important functions, the immune system consumes considerable bioenergy. Rationally allocating energy materials for immune operation requires precise regulation of the metabolic pathways of immune cells. The study of immune metabolism can be traced back to a century ago when it was observed that immune cell metabolism is highly dependent on glucose (Levene and Meyer, 1912). The study of the immune metabolism of neutrophils and T cells began in the 1950s and 1960s. In the 1950s, studies found that neutrophils primarily activate respiratory bursts through glycolysis (Valentine and Beck, 1951; Sbarra and Karnovsky, 1959). In the 1960s and 1970s, glycolysis and glutamine breakdown were reported to be important in T cell activation, and mitotic lectins were observed to significantly stimulate the uptake of glucose and glutamine by lymphocytes (Parenti et al., 1966; Polgar et al., 1968; Roos and Loos, 1973; Hume et al., 1978). During the gradual exploration of metabolites, it was discovered that immune cells use metabolites for two important purposes: providing a substrate for ATP synthesis to maintain the activated state of the immune system and synthesizing macromolecular substances, such as DNA, RNA, protein, and cell membrane, for the immune system to function (Ganeshan and Chawla, 2014). Glucose is the primary substance involved in the energy metabolism of immune cells with lipids providing part of the energy. When immune requirements increase, T cells require extremely high levels of glycolysis. T cells express a large number of GLUT family members with the main participants including the kinase mammalian target of rapamycin complex 1 (mTORC1), transcription factor c-Myc, and hypoxia-inducible factor 1α (HIF-1α), thereby obtaining a large amount of energy for differentiation (Shi et al., 2011; Wang R. et al., 2011; Sinclair et al., 2013; Macintyre et al., 2014). The mechanism involves metabolic reprogramming (Sinclair et al., 2013). The difference is that the levels of glycolysis in Treg cells are relatively low, and fatty acid oxidation provides the primary fuel (Michalek et al., 2011). Further metabolic programming of memory T cells also depends on downregulation of glycolysis levels and conversion to lipid oxidation (Gubser et al., 2013). B cells primarily receive B cell receptor (BCR) stimulation, TLR4 stimulation, and IL-4 stimulation but not mTORC1 stimulation (Doughty et al., 2006; Dufort et al., 2007; Caro-Maldonado et al., 2014). Under these stimulatory conditions, B cell glycolysis or OXPHOS levels are upregulated, but the magnitude is not as high as that of T cells (Donnelly and Finlay, 2015). Aerobic glycolysis increases levels of the abovementioned immune cells to varying degrees, which not only promotes the biosynthesis of these cells but also has important significance for controlling cell differentiation and regulating effectors (Donnelly and Finlay, 2015). Lipids are not the primary functional source, but their participation in immune metabolism has attracted increasing attention. For example, the maintenance of immune cell quiescence, anti-inflammatory alternative macrophage activation, and CD8 memory formation is highly dependent on the β-oxidation of fatty acids and mitochondrial oxidative metabolism (Vats et al., 2006; Odegaard et al., 2007; Pearce et al., 2009). Despite rapid progress over the past century, our understanding of immune metabolism is still in its infancy. Limitations include incomplete identification of metabolic effectors in the immune cell bank, lack of understanding of inducers and sensors that activate immune cells, lack of consideration of the diversity of immune cell spectrum and tissue specificity, etc., (Ganeshan and Chawla, 2014). In the future, immune metabolism research will enhance or weaken the lethality of the immune system by changing the metabolic program of immune cells and provide us with treatment options for cancer and infections. This article elaborates on the role of LDs in immune cells. For innate immunity, we will focus on the role of LDs in neutrophils, mast cells, macrophages and dendritic cells. For specific immunity, we will focus on the interaction between LDs and T cells.

## Lipid Droplets and Immune Cells

### Lipid Droplets and Neutrophils

Neutrophils, derived from bone marrow stem cells, are part of the innate immune system. Known as polymorphonuclear granulocytes (PMNs) due to their lobulated nuclei, they are the most numerous white blood cells in the blood and are characterized by a short life, fast renewal, and high quantities. Neutrophils exert chemotactic, phagocytic, bactericidal, and other biological functions, which are primarily related to the many fine neutral particles evenly distributed in their cytoplasm. During acute inflammation, in addition to phagocytosis and killing bacteria, neutrophils can also perform chemotaxis and recruit other effector cells to the infected site through the synthesis and secretion of cytokines or the mechanism of mutual contact, regulating innate or adaptive immunity and further enhancing the killing effect of bacteria. In recent years, the role of neutrophils in addition to acute inflammation has attracted increasing attention. Interestingly, the cytokines released by neutrophils are primarily lipid mediators that include arachidonic acids. Therefore, the metabolic requirements of neutrophils have received increasing attention. However, compared to macrophages and T cells, there are few studies examining the metabolic characteristics of neutrophils, particularly lipid metabolism. Regardless of the type of immune cell, lipid metabolism primarily occurs in the mitochondria; however, it is worth noting that neutrophils have almost no mitochondria and perform glycolysis to produce energy in most cases.

In mammals, as a dynamic organelle that is attracting increasing attention, LDs are closely related to mitochondria. This association may be controlled by AMPK because AMPK activation causes LDs to spread across stable microtubules, which is thought to increase their interaction with mitochondria (Herms et al., 2015). Several recent studies have demonstrated that LDs are involved in various biological processes, particularly innate immunity. Therefore, it is not difficult to speculate that LDs play an extremely important role in the occurrence, development, and function of neutrophils.

A remarkable phenomenon observed during bone marrow differentiation into neutrophils is the accumulation of LDs (Inazawa et al., 2015). The formation of LDs or adipogenesis is an important part of neutrophil differentiation. LD formation is not only related to lipogenesis but is also closely related to fat degradation, all of which have been shown to affect the differentiation of neutrophils. Inhibition of galectin-12 in ATATA-induced human promyelocytic leukemia cell line NB4 inhibits lipid droplet formation *via* PPARγ and ultimately promotes the differentiation of neutrophils (Xue et al., 2016). Expression levels of PPARγ, p-CREB, C/EBPα, and C/EBPβ in galectin-12 knockdown cells are lower, all of which regulate lipid production (Xue et al., 2016). Notably, PPARγ is a key transcription factor that regulates adipogenesis, stimulates lipid production, and induces NB4 cell differentiation (Yasugi et al., 2006). In addition to adipogenesis, neutrophil differentiation is also closely related to fat degradation, which was recently confirmed in a study examining the mechanism of autophagy in neutrophil differentiation (Riffelmacher et al., 2017). The results of this experiment demonstrated that neutrophils deficient in Atg7 (an essential autophagy protein) exhibit increased glycolytic activity, impaired mitochondrial respiration, reduced ATP production, impaired differentiation, decreased lipolysis, and accumulation of LDs (Riffelmacher et al., 2017). Autophagy is closely related to centrifugal differentiation and metabolic conversion. During the process of normal neutrophil differentiation, autophagy can degrade LDs primarily by targeting lipids to lysosomal lipase to hydrolyze phagosomes. On the one hand, this prevents lipid accumulation and avoids lipotoxicity. On the other hand, the degraded LDs release free fatty acids to maintain the total amount of cytosolic fatty acids. Normal neutrophils undergo metabolic reprogramming from glycolysis to FFA-dependent oxidative phosphorylation during their development, and autophagy can decrease the levels of glycolysis in neutrophils and increase the levels of oxidative phosphorylation (Riffelmacher et al., 2017). In addition to autophagy, a dynamic hub of cellular lipid metabolism, LDs can isolate excess lipids and provide a source of FAs through LD-related neutral lipases, for example, ATGL. LD-related neutral lipase can respond to the metabolic state of cells, degrading TAG to produce abundant free FAs, which can be transferred to the mitochondria to produce a large amount of ATP to meet physiological needs. During the normal differentiation of neutrophils, lipid metabolism is closely related to cellular differentiation. However, the specific mechanism by which lipid metabolism, including lipidogenesis and lipolysis, affects neutrophil differentiation remains to be further studied.

As a key marker of leukocyte activation, the polymorphism of LDs during inflammation is well known, and their biogenesis is cell-type and stimulus dependent (Walther and Farese, 2012). When cells are activated, the number and size of LDs in neutrophils rapidly increase, mainly to prepare for the secretion of cytokines (Bozza and Bandeira-Melo, 2005). LDs can store the precursors of cytokines, primarily arachidonic acids, in neutrophils. Arachidonic acids primarily include prostaglandins, thromboxanes, and leukotrienes, which are derived from arachidonic acid (AA) and the related 20-carbon polyunsaturated fatty acid (Bozza et al., 2011). Free AA can be obtained by enzymatic decomposition of membrane phospholipids or can be released from AA-rich triglycerides stored in LDs in some cells. LDs modulate the immune response in neutrophils, as impaired LD lipolysis is accompanied by reduced arachidonic acid release (Schlager et al., 2015). Since arachidonic acid is a non-storable mediator that is rapidly formed and released after cellular stimulation, the release intensity of bioactive lipid mediators in neutrophils depends on the amount of LDs. Prostaglandin E2 (PGE2), the most important bioactive mediator secreted by neutrophils, accelerates blood flow and produces oedema and pain, and its synthesis is primarily related to AA, which is related to LDs (Kawahara et al., 2015). As a key signaling molecule in lipid metabolism in neutrophils, AA acts as a second messenger to transmit signals in cells and can also be converted into prostaglandin E2 by a variety of enzymes. During prostaglandin E2 synthesis, three main enzymes are involved: phospholipase A2 (PLA2), cyclooxygenase (COX-1 and COX-2), and terminal prostaglandin synthase. Notably, phospholipids can be cleaved by phosphorylated cytoplasmic PLA2 to produce free fatty acids, including AA, which in turn forms prostaglandins G2 (PGG2), PGH2, and PGE2, catalyzed by cyclooxygenase (COX-1 and COX-2) and terminal prostaglandin synthase (Gupta and Dubois, 2001; Bapna and Chauhan, 2015). Cr-LAAO is an L-amino acid oxidase isolated from venom that stimulates neutrophil activation and arachidonic acid production (Pontes et al., 2014). During the activation of neutrophils induced by Cr-LAAO, the microarray data also demonstrated that the expression of lipid-related genes was upregulated not only due to signal transduction and metabolism genes, such as DGAT1, DGAT2, cPLA2-α, and COX-1. COX-2 and prostaglandin E synthase, as well as others, also include genes related to lipid droplet formation, including PLIN2, PLIN3, DGAT1, and DGAT2 (Paloschi et al., 2020). Perilipin 3 (PLIN3) plays a vital role in the formation of LDs in neutrophils and in the release of PGE2 because when PLIN3 is downregulated by siRNA treatment, LDs in HL-60-derived neutrophils disappear, the secretion of PGE2 levels decreases by 65%, and the enzymes involved in the synthesis of PGE2 are also inhibited (Nose et al., 2013).

**FIGURE 2:**
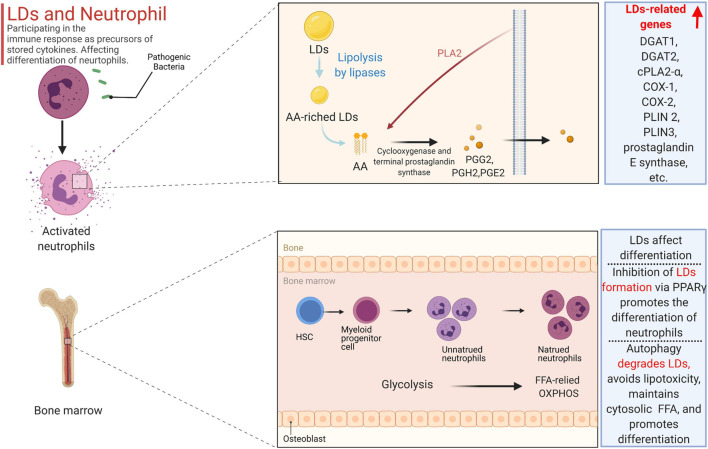
The formation of LDs is not only related to lipogenesis but also closely related to fat degradation, all of which not only affects neutrophil differentiation but also participates in the immune response as precursors of stored cytokines (primarily arachidonic acid) in the neutrophil. Free AA can be obtained by enzymatic decomposition of membrane phospholipids or can be released from AA-rich triglycerides stored in LD in some cells. Phospholipids can be cleaved by phosphorylated cytoplasmic PLA 2 to produce free fatty acids including AA, which in turn forms prostaglandins G2 (PGG 2), PGH 2, and PGE2, catalyzed by cyclooxygenase (COX-1 and COX-2) and terminal prostaglandin synthase. In the activation of neutrophils induced by Cr-LAAO, the microarray data also proved that the expression of lipid-related genes was up-regulated. Inhibition of galectin-12 in ATATA-induced human promyelocytic leukemia cell NB4 inhibits lipid droplet formation *via* PPARγ, and ultimately promotes the differentiation of neutrophils. Autophagy can degrade LD, not only preventing lipid accumulation and avoiding lipotoxicity, but also releasing free fatty acids to maintain the total amount of cytosolic fatty acids.

Overall, the formation of LDs is not only related to lipogenesis but is also closely related to fat degradation, all of which not only affects neutrophil differentiation but also participates in the immune response as precursors of stored cytokines (primarily arachidonic acid) in neutrophils (Figure 2).

### Lipid Droplets and Mast Cell

Mast cells, derived from myeloid stem cell precursors, play a key role in the initiation, maintenance, and resolution of the inflammatory response and are primarily involved in hypersensitivity and antiparasitic infection *in vivo*. After activation, mast cells release a variety of cytokines and chemokines, including histamine, serotonin, and arachidonic acid, playing a wide range of pathophysiological roles in a variety of diseases, including atherosclerosis, rheumatoid arthritis, and asthma (Abraham and St John, 2010; Galli and Tsai, 2012). Mast cells can be divided into two types: mucosal cells, primarily distributed in the submucosa with proliferation dependent on T cells, and connective tissue mast cells, primarily distributed in the connective tissues around subcutaneous small blood vessels and independent of T cells. Mast cells are phenotypically and functionally heterogeneous, depending on the microenvironment in which they mature. There are two main ways mast cell undergo activation: PPR-mediated and IgE-mediated. The PRR on the surface of mast cells recognizes the PAMPs of pathogenic microorganisms and then initiates the activation signal. In addition, mast cells express high-affinity IgE Fc receptors (FcεRIs) on their surface that can be activated by corresponding ligands. Mast cells contain two specialized proinflammatory organelles: secretory granules containing histamine, serotonin, and matrix active protease and cytoplasmic liposomes, which are the sites of arachidonic acid metabolism, precursor storage, and synthesis in mast cells (Galli and Tsai, 2010; Melo et al., 2011). When mast cell FcεRIs cross-link with each other through the phosphorylation of ITAM at the C-end of the γ chain mast cells are activated to perform physiological functions. On the one hand, the phosphorylation of ITAM activates SYK and Fyn tyrosine kinase and activates the PI-PLCγ signal chain to phosphorylate the light chain of myosin in the cytoplasm, leading to degranulation and release of biologically active mediators. Activated mast cells cannot exert a direct killing effect on pathogenic microorganisms entering the body, but they can quickly degranulate, release inflammatory factors in the granules into surrounding tissues, recruit immune effector cells, such as neutrophils and basophils, to reach the site of infection, and initiate a local inflammatory response. On the other hand, phosphorylation of ITAM activates the mitogen promoter protein (MAK) kinase signaling pathway, decomposing membrane phospholipid choline (PC) to produce arachidonic acid, which then passes through the cyclooxygenase and lipoxygenase pathways to synthesize prostaglandin D2 and leukotrienes and decompose alkylated phospholipids to generate LYSO-PAF, which is converted into platelet-activating factor (PAF) through the action of acetyltransferase. Prostaglandin D2, leukotriene, and platelet-activating factors are lipid mediators, collectively known as arachidonic acids, which induce differential biological effects. The activation of mast cells and PGD2 secreted by mast cells can affect the activation of leptin-induced eosinophils in humans because studies have shown that leptin-induced eosinophils in sensitized BALB/c mice depends on PGD 2 derived from mast cells (Amorim et al., 2020). The accumulation of liposomes in mast cells was associated with increased levels of leukotriene synthase (LTC4S and 5-LO) because studies have shown that insulin-containing adipogenesis stimulators can inhibit the degranulation potential of mast cells and upregulate the biogenesis of liposomes and the secretion of eicosanoids (Greineisen et al., 2012). As TGs in cytoplasmic LDs are the primary storage sites of AA and because ATGL can hydrolyze the AA-containing TG that exists in the LD of human mast cells, silencing of ATGL expression can lead to a significant accumulation of neutral lipids in the cytoplasm of mast cells and consequently reduce the production of eicosanoids by these cells. In addition to storing lipids, the accumulation of LDs in mast cells can change the dynamics of calcium signals, accelerating the spread of calcium ions, which affects the intensity and characteristics of the calcium response after antigen exposure stimulation (Greineisen et al., 2014). There is a close physical and functional interaction between liposomes and mitochondria (Klecker et al., 2017; Valm et al., 2017; Gemmink et al., 2018). The lipid droplet-associated proteins Perilipin 5 and SNAP23 are reported to play a crucial role in the physical and metabolic connections between liposomes and mitochondria (Wang H. et al., 2011). Chen et al. (2016) used soft X-ray tomography (SXT) combined with fluorescence microscopy to study the detailed structure of organelles during mast cell activation and observed granular fission, granular-cell membrane fusion, and small vesicles sprouting from the granules. Mast cell degranulation involves multiple membrane events, including particle fusion and fission and particle and membrane fusion, and these processes require intracellular calcium signaling and mitochondrial function. LD biogenesis by mast cells in the body is not only closely related to the ER but is also affected by hormones in the body, especially insulin. In RBL2H3, a reliable model of mast cells, exposed to insulin for a long time, liposomes excessively accumulate in the cell, and various markers of UPR (IRE1α, phosphorylated PERK, and ATF6) are upregulated, leading to the ER response. At the same time, the expression of proteins related to autophagy, such as ATG3, ATG12, ATG7, Beclin, and LC3A, has also been observed to increase accordingly (Greineisen et al., 2015).

**FIGURE 3:**
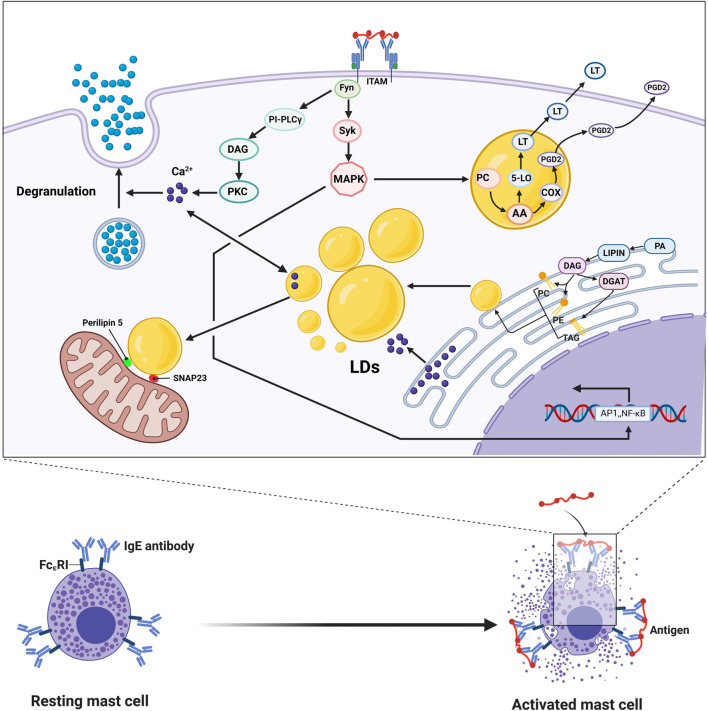
IgE cross-linking mediated activation of mast cells leads to degranulation and release of large amounts of bioactive lipids, including prostaglandin D2 and leukotriene (LT). After FcεRI is cross-linked with each other, the phosphorylation of ITAM activates Fyn and Syk, which in turn mediates activation of PKC through the PI-PLCγ signaling chain, resulting in increased intracellular calcium concentration and phosphorylation of the intracytoplasmic myosin light chain, leading to degranulation and release of bioactive mediators. Phosphorylation of ITAM activates the mitogen-activated protein (MAK) kinase signaling pathway, causing the breakdown of phospholipid choline (PC) in the presence of phospholipase A2 to produce arachidonic acid (AA), which is then combined into PGD2 and LT, respectively, *via* the cyclocythase and lipoxygenase pathways. Activation of MAPK also increases the transcription of several nuclear transcription factors, such as NF-κB and AP1. After FcεRI is cross-linked, LDs are not only the source of calcium ions in mast cells but also the sinks of calcium ions, acting as the source and absorber of calcium ions, thereby affecting the corresponding signal pathways. The LDs-associated proteins Perilipin 5 and SNAP23 play crucial roles in the physical and metabolic connections between LDs and mitochondria. The synthesis of LDs is mainly carried out in the endoplasmic reticulum, accompanied by the synthesis of TAG, PC, and PE with FA as the substrate under the action of various enzymes.

In general, the role of lipid droplets in mast cells is primarily to act as the site of metabolism, storage, and synthesis of lipid inflammatory mediators and to change the dynamics of calcium signaling to affect the degranulation of mast cells and the secretion of inflammatory mediators (Figure 3).

### Lipid Droplets and Macrophages

In general, having a very heterogeneous cell population that exhibits unique phenotypes and functions in the complex microenvironment of the body, macrophages are the hub that connects innate and adaptive immunity in the body. In contrast to monocytes in the blood after passing through blood vessels, macrophages are derived from precursor cells in the bone marrow. They exist throughout the body and participate in the inflammatory response as the blood circulates throughout the body. Although the morphology and function of macrophages are heterogeneous, which is determined by the macrophage microenvironment composed of a variety of cytokines, cells, pH, and oxygen content, their main function is to participate in the phagocytosis of various substances in the body, such as bacteria, viruses, fungi, parasites, cell debris, and tumor cells, participating in innate immunity and, at the same time, as antigen-presenting cells that absorb, process and present antigens to T cells, participating in adaptive immunity. Depending on their phenotype and function, macrophages can be roughly divided into two categories: M1 and M2. M1 type macrophages induced by tumor necrosis factor (TNF)-α, interferon (IFN)-γ) or invading pathogens secrete chemotactic and proinflammatory cytokines (IL-1β, TNF-α, and IL-12), present antigens, participate in Th1-mediated positive immune response and perform immune surveillance; M2-type macrophages have only weak antigen-presenting ability and play an important role in Th2-dependent immune regulation by secreting inhibitory cytokines, such as IL-10 and TGF-B, to downregulate the immune response (Mehla and Singh, 2019).

In recent years, multiple studies have shown that LDs and macrophages are closely related to diverse functions in different microenvironments, such as atherosclerosis, tumor microenvironments, and infection (parasites, viruses, bacteria, and fungi).

**FIGURE 4:**
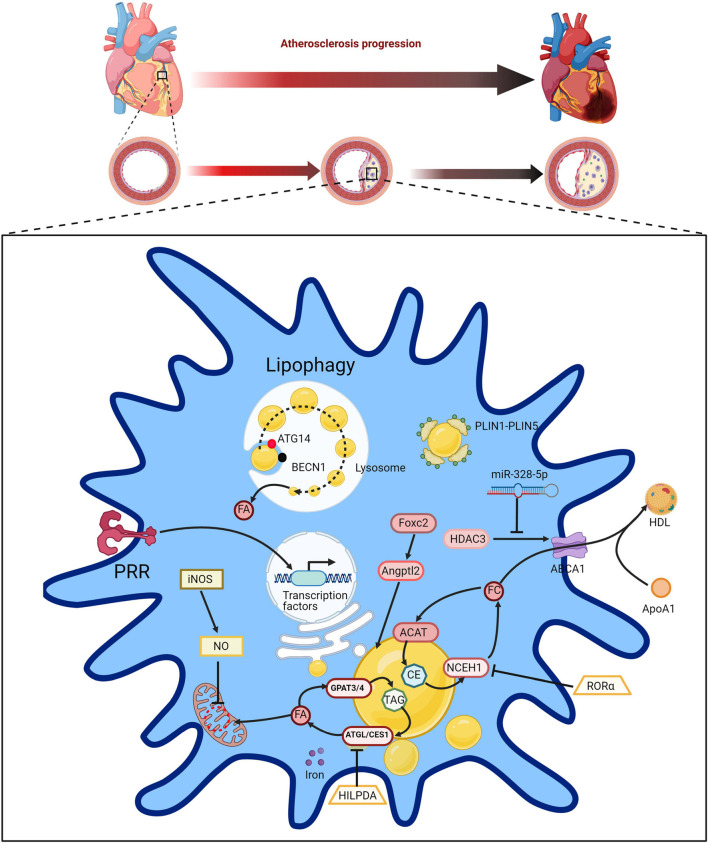
Atherosclerosis is accompanied by the accumulation of LDs in macrophages. Macrophages can reduce excessive lipid accumulation in the body by increasing the lipopolysis of intracellular LDs and avoiding the damage of cells from lipid toxicity, thus alleviating the further development of atherosclerosis. Intracellular free cholesterol (FC) can be transferred extracellular by ABCA1 to combine with extracellular apolipoprotein A1 (ApoA1) to form high-density lipoprotein (HDL). The activity of ABCA1 is mediated by miR-328-5p reduction regulated histone deacetylase 3 (HDAC3). TAGs can be synthesized by FA under the action of GPAT3 and GPAT4, and TAGs break down to produce free fatty acids under the action of ATGL and CES1. The hydrolysis of cholesterol ester (CE) is catalyzed by neutral cholesterol ester hydrolase 1 (NCEH1) to produce free cholesterol (FE), and the synthesis of CE is catalyzed by acyl-coenzyme A-cholesterol acyltransferase (ACAT). Foxc2-induced Angptl2-mediated lipid accumulation is also associated with atherosclerosis. Nitric oxide (NO) derived from iNOS can inhibit mitochondrial respiration. The lipophagy can be mediated by BECN1 and ATG14 in macrophages, decomposing LDs to produce FA. The perilipin family of PLIN1-PLIN5 is the mark in LDs accumulation in macrophage. Hypoxia-inducible lipid droplet-associated protein (HILPDA) is a physiological inhibitor of ATGL-mediated lipopolysis in macrophages, binding to the intracellular triglyceride hydrolase ATGL and inhibiting ATGL-mediated triglyceride hydrolysis. Retinoic acid receptor-related orphan receptor α(RORα) can induce the high expression of NCEH1 in macrophages, thereby promoting the hydrolysis of cholesterol ester in LDs and alleviating lipid accumulation.

Macrophages can reduce excessive lipid accumulation in the body by increasing the lipolysis of intracellular LDs, avoiding damage to cells from lipid toxicity and alleviating the further development of atherosclerosis. The primary mechanisms include the miR-328-5p reduction-mediated histone deacetylase 3/ATP-binding cassette transporter A1 pathway (Huang et al., 2021), lipid droplet ubiquitination mediated by AUP1 (Robichaud et al., 2021), TAG synthesis guided by GPAT3 and GPAT4 (Quiroga et al., 2021), hydrolysis of cholesterol ester catalyzed by neutral cholesterol ester hydrolase 1 (Matsuoka et al., 2020), Foxc2-induced Angptl2-mediated lipid accumulation (Yang L. et al., 2020), inhibition of mitochondrial respiration by iNOS-derived nitric oxide (Rosas-Ballina et al., 2020), SphK2-affected lipid droplet decomposition mediated by autophagosomes and lysosome (Ishimaru et al., 2019), BECN1 and ATG14 mediated autophagy (Singh et al., 2009; Ouimet et al., 2011; Hadadi-Bechor et al., 2019), lipid accumulation marked by the perilipin family of PLIN1-PLIN5 (Sztalryd and Brasaemle, 2017; Bosch et al., 2020), LD biogenesis mediated by PPAR signaling pathway (Yang T. et al., 2020), acute iron deprivation (Pereira et al., 2019), neutral lipase-mediated hydrolysis of neutral fat in LDs (van Dierendonck et al., 2020), and fat phagocytosis mediated by Hmgb1, Hmgb2, Hspa5 and Scarb2 (Robichaud et al., 2021; Figure 4). HILPDA is a physiological inhibitor of ATGL-mediated lipolysis in macrophages that binds to the intracellular triglyceride hydrolase ATGL and inhibits ATGL-mediated triglyceride hydrolysis (van Dierendonck et al., 2020). Retinoic acid receptor-related orphan receptor α induces high expression of NCEH1 in macrophages, promoting the hydrolysis of cholesterol ester in LDs and alleviating lipid accumulation (Matsuoka et al., 2020). In addition, MLX transcription factors and LDs can bind to each other on the surface of LDs, which can alter the storage level of LDs in macrophages to meet the metabolic needs of cells in the current microenvironment (Mejhert et al., 2020). When there are too many LDs, it can weaken the metabolic pathway that uses glucose as the substrate, and when LDs are too low, MLX can regulate metabolic genes such as AQP3 and increase the storage of LD (Mejhert et al., 2020). Macrophage autophagy can enhance lipid decomposition by lipohydrolase and reduce LD levels, alleviating some metabolic diseases related to elevated lipid levels, such as atherosclerosis. Interestingly, recent studies have shown that macrophage autophagy appears to be heterogeneous, and different lipid substrates or different inflammatory activation modes may lead to differences in the levels of macrophages processing LDs (Hadadi-Bechor et al., 2019).

Compared to macrophages in normal tissues, tumor-associated macrophages (TAMs) located in the tumor microenvironment various distinct characteristics and functions, including inhibiting antitumor immunity and promoting tumor cell proliferation, transformation, escape, and metastasis (Conway et al., 2016). Cancer cells tend to release fatty acids into the surrounding environment to form a fatty acid-rich tumor microenvironment. Different types of TAMs located in this environment have different metabolic pathways. Glycolysis of proinflammatory macrophages increases; however, anti-inflammatory macrophages, with increased FAO mediated by PPARγ, PGC-1β, and STAT6, exhibit the characteristics of M2 phenotype macrophages and have a strong ability to inhibit antitumor immunity and promote tumorigenesis and development (Mehla and Singh, 2019). LDs play a very important role in the formation of the anti-inflammatory phenotype of TAMs, regulating lipid synthesis and metabolism, and the free FAs produced by metabolism can be used as a substrate for mitochondrial productivity, thereby regulating oxidative phosphorylation of mitochondria (Boeszoermenyi et al., 2015). Of note, the phenotype of tumor-associated macrophages is not static. M1 and M2 subtypes can be interconverted. Considering the proinflammatory characteristics of M1 macrophages and the tumor-promoting characteristics of M2 macrophages, we hope to improve the tumor microenvironment and promote the transformation of tumor-related macrophages from the M2 type, which suppresses antitumor immunity, to the M1 type, which promotes the inflammatory response. Studies have shown this possibility. Bose et al. (2019) demonstrated through RAW264.7 cells that TGF-β induces the formation of LDs in tumor-associated macrophages, and the transformation of M2 macrophages to M1 macrophages was observed when TGF-β-induced LDs were inhibited. Further experiments showed that this may be related to the MEK1/2 axis (Bose et al., 2019). It is not difficult to see that the M2 type of TAM FAO has a strong demand, so targeting the PPARγ, PGC-1β, and STAT6 signaling pathways to reduce their mediated FAO is likely to be an effective strategy for promoting the transformation of M2 macrophages to M1 macrophages (Mehla and Singh, 2019).

In addition, macrophages are involved in the body’s immunity against infection. LDs, the accumulation of which is a sign of infection, also play a very important role in this process. However, there is still much controversy over whether LDs promote the body’s resistance to infection or support the body’s susceptibility to infection. Previous views have held that LDs support infection primarily by providing fatty acids as substrates of FAO to produce large amounts of ATP for bacteria and providing pathogenic microorganisms with lipids to synthesize their membrane structure to ensure the normal structure and function of their membrane structure, promoting infection persistence and further development (Allen and Martinez, 2020). Studies have shown that *Mycobacterium tuberculosis* can persist in the body, which is closely related to the activation state and metabolic pathways of its host cells (Genoula et al., 2020). In particular, the results of this study indicated that the continuous accumulation of LDs mediated by STAT6 in foam macrophages (a type of macrophage rich in LDs is a sign of continuous *Mycobacterium tuberculosis* infection) is beneficial to tuberculosis. The long-term existence of *Mycobacterium tuberculosis* in the body promotes the further development of infection (Jaisinghani et al., 2018; Guerrini and Gennaro, 2019; Genoula et al., 2020). The accumulation of LDs is related to host-pathogen interactions. NR1D2-mediated PNPLA2/ATGL inhibition disrupts the lipid metabolism of macrophages in the human body, leading to lipid accumulation and weakening the effect of autophagy on *Burkholderia pseudomallei* immunity to infection, leading to the continued development of infection (Tang et al., 2021). In *Mycobacterium tuberculosis*, the interaction between LDs and pathogen-containing phagosomes is controlled by mycobacterial cell wall components (LAM and PIM) and Rab7 (Roque et al., 2020). However, the results of a recent study indicated that to deal with infections, LDs in mammals assemble antibiotics and immune proteins to form a complex that fights pathogens and destroys them (Bosch et al., 2020). This mechanism is not unique to immune cells such as macrophages but also occurs in other cells in the body (Bosch et al., 2020). Through gene interaction analysis, the experimental results showed that immune proteins that can fight pathogens primarily gather around LD surface protein 2 (PLIN2) (Bosch et al., 2020). Therefore, the anti-infection effect of LDs on the body and the specific mechanism needs to be further studied and elucidated.

Overall, the interaction between LDs and macrophages is heterogeneous, which means that they exhibit different functions in different microenvironments, such as atherosclerosis, tumor microenvironments, and infection. Macrophages can reduce excessive lipid accumulation in the body *via* multiple mechanisms to alleviate the further development of atherosclerosis. LDs play a very important role in the formation of the TAM anti-inflammatory phenotype (M2) and are a potentially effective target for promoting the transformation of tumor-related macrophages from M2 to M1. Macrophages are also involved in the body’s immunity against infection, but whether LDs promote the body’s resistance to infection or support infection remains controversial.

### Lipid Droplets and Dendritic Cells

Dendritic cells (DCs) originate from pluripotent hematopoietic stem cells in the bone marrow and are widely distributed throughout the body, but their concentrations are quite low. Representing the link between adaptive and innate immunity, the most important function of DCs is to absorb, process, and present antigens, stimulating the body to produce an immune response and regulating the body’s immune response. According to phenotype and function, DCs can be divided into conventional DCs (cDCs) and plasmacytoid DCs (pDCs). Conventional DCs primarily release IL-12 after being stimulated with bacterial components and mainly express TLR2, TLR3, TLR4, and TLR5, and their main function is to induce a specific immune response against invading antigens and maintain self-tolerance (Chistiakov et al., 2019). Loss of rapamycin complex 2 (TORC2) leads to the proinflammatory phenotype of cDCs and increases the stimulation of T cells (Watson et al., 2019). The primary function of pDCs is to produce large amounts of type I interferon against microorganisms, especially viral infections, and to stimulate corresponding T cell responses. Under the stimulation of unmethylated CPG motifs derived from viruses and bacteria, pDC pits produce high levels of type I interferons, especially IFN-α, through the TLR7/9 signaling pathway, which directly interferes with viral replication and activates mononuclear macrophages to kill pathogenic microorganisms (Hedrick, 2015; Chistiakov et al., 2015b). DCs can absorb antigens through receptor-mediated endocytosis, macrophage cytosis, and phagocytosis, as well as process antigens in cells to clarify the pathogenic microorganisms, products, and harmful antigenic substances that invade the body. Through antigen presentation, DCs directly or indirectly regulate T and B cells, which primarily depends on costimulatory molecules, adhesion molecules, and antigen peptide/MHC molecular complexes on the surface of DCs, as well as secreted cytokines.

**FIGURE 5:**
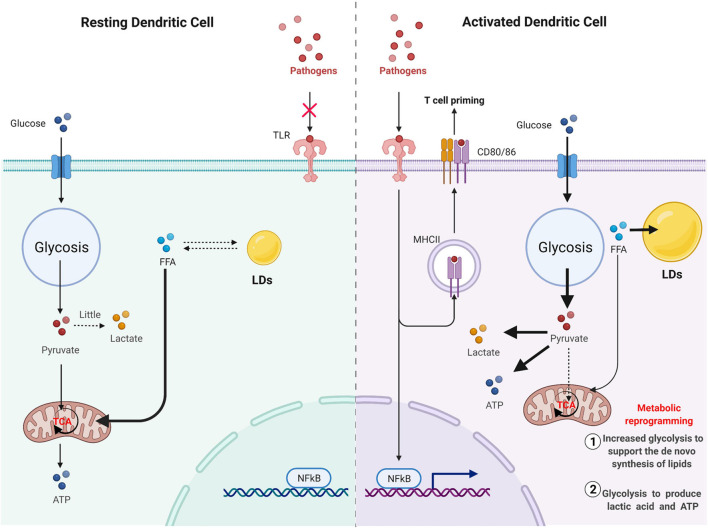
Pathogen invasion is often accompanied by the accumulation of LD. Resting DC mainly uses free fatty acids as substrates for oxidative phosphorylation through fatty acid oxidation to generate energy to meet physiological needs. Glucose can also be used as a substrate to produce energy in resting DC, mainly because glucose can be converted to pyruvate by glycolysis, and then enter the mitochondrial TCA cycle. However, when DC cells are activated, pyruvate produced by glycolysis will no longer enter TCA and will be converted to lactic acid capacity under a harsh environment such as hypoxia. DC activation mediated by TLR will eventually lead to the accumulation of LD, mainly due to metabolic reprogramming. This metabolic change process can be divided into the first stage of increased glycolysis to support the *de novo* synthesis of lipids and the second stage of glycolysis to produce lactic acid and ATP.

In immune cells, LDs are considered structural markers of inflammation (Bozza et al., 2007). Pathogen invasion is often accompanied by the accumulation of LDs (Figure 5). When host cells recognize pathogens through PRR, especially antigens through the TLR family, LDs accumulate in immune cells. This accumulation of LDs not only reflects the excess energy in DCs but also suggests to a certain extent that there is metabolic reprogramming in DCs. Resting DCs primarily use free fatty acids as substrates for oxidative phosphorylation through fatty acid oxidation to generate energy to meet physiological needs. Glucose can also be used as a substrate to produce energy in resting DCs, mainly because glucose can be converted to pyruvate by glycolysis and then enter the mitochondrial TCA cycle (Krawczyk et al., 2010). However, when DCs are activated, their metabolic mode markedly changes, becoming dominated by an aerobic glycolysis capacity similar to that of tumors, which is called the Warburg effect; that is, pyruvate produced by glycolysis no longer enters the TCA and is instead converted into lactic acid under harsh environments, such as hypoxia (Kelly and O’Neill, 2015). In cDCs, mTORC2-deficient DCs exhibited enhanced glycolysis and a proinflammatory phenotype and enhanced T cell activation (Watson et al., 2019). DC activation mediated by TLRs eventually leads to the accumulation of LDs, primarily due to metabolic reprogramming. This metabolic change process can be divided into the first stage of increased glycolysis to support the *de novo* synthesis of lipids and the second stage of glycolysis to produce lactic acid and ATP (Everts et al., 2014). Accumulation of citrate in DCs and the biosynthesis of prostaglandin are landmark events that occur during the metabolic reprogramming of DCs activated by TLR (O’Neill, 2014).

Lipid Droplets in normal immune cells seem to be related to immune enhancement (Bosch et al., 2020). For example, liposomes promote cross-presentation through phagocytosed antigens by MHC class I in DCs (Bougneres et al., 2009). However, in tumors, the increased intracellular lipid content of tumor–infiltrating DCs is observed more often (Yin et al., 2020). In addition, tolerable lipid DCs have been found in malignant microenvironments (Herber et al., 2010). Thus, LDs in DCs cannot be generally considered proinflammatory or anti-inflammatory because the function of LDs is primarily determined by lipid-associated proteins and internal lipids, which are closely related to the local microenvironment. The tumor microenvironment (TME) is characterized by hypoxia, nutritional deficiencies, reactive oxygen or nitrogen species, and elevated levels of adenosine, lactic acid, and immunosuppressive factors such as IL-10 and PD-L1 (Veglia and Gabrilovich, 2017). The cross-antigen presentation of DCs in the tumor microenvironment is weakened (Herber et al., 2010), but the specific mechanism whereby this occurs remains unclear. However, multiple factors are related to this phenomenon. The harsh local microenvironment impairs endoplasmic reticulum folding in cancer cells and tumor-infiltrating DCs, leading to ER stress, which is closely related to the accumulation of LDs in DCs. In this case, ER stress may be an intermediate pathological process induced by LDs in cancer immune cells. Silencing of the endoplasmic reticulum stress connector XBP-1 in cancer-associated dendritic cells results in a reduction in the number and size of LDs in each cell, improved immune capacity, antitumor effects, and ultimately prolonged survival (Cubillos-Ruiz et al., 2015). Tumor cells use a variety of signaling molecules to communicate with DCs (DeVito et al., 2019). Exosomes are an important part of the tumor microenvironment and can play a role in signal transduction between tumor cells and non-tumor cells. Recent studies have shown that, compared to normal exosomes, the FA content of exosomes in the tumor microenvironment is increased, which can lead to LD accumulation in DCs and upregulation of mitochondrial FAO through activation of PPARα, resulting in the metabolic transition to mitochondrial oxidative phosphorylation and driving immune dysfunction in DCs (Yin et al., 2020). In addition to exosomes, tumor cells can also utilize paracrine Wnt5a/β-catenin signaling to activate PPARγ in DCs, enhance FAO and lead to DC dysfunction (Ramakrishnan et al., 2014). Mesothelioma cells exposed to acidosis in the tumor microenvironment promote the secretion of TGF-β2, and TGF-β2-dependent LDs accumulate in metabolic reprogramming DCs (Trempolec et al., 2020). Saponin-based adjuvants (SBAs) can induce intracellular liposomes in a subset of CD11b+ DCs, increasing the antigen presentation of DC cells (den Brok et al., 2016). Oxidized lipids prevent cross-presentation by reducing the expression of MHC class I peptide complexes on the cell surface, and the accumulation of non-oxidized lipids does not affect cross-presentation (Ramakrishnan et al., 2014). By combining lipidomics and molecular dynamics simulations, Veglia et al. (2017) found that liposomes with DC accumulation in tumor-carrying hosts contain electrophilic oxidized trapping (ox-TR) lipids that covalently bind to heat shock protein 70, and this interaction prevents pMHC transport from late endosomal/lysosomal transport to the cell surface. Oxidized lipids only affect the surface level of pMHC-1 with exogenous but not endogenous peptides (Ramakrishnan et al., 2014).

In addition to tumors, the relationship between LDs and DCs also plays a key role in the occurrence and progression of diseases in other diseases. Hypoxia in the presence of obesity induces the activation of the HIF1α transcription factor, which can induce lipid accumulation in DC cells, inhibiting CDC activation, promoting an anti-inflammatory phenotype, and leading to the formation of atherosclerotic plaques and the enhancement of adipose tissue inflammation (Wood et al., 2020). Mouse bone marrow-derived DCs can be activated by the flagellum of Leishmania protozoa and undergo metabolic reprogramming to induce lipid accumulation (Lecoeur et al., 2013). Allithiamine has been identified as a coactivator of the PDH complex (PDC), which promotes the conversion of cytosolic pyruvate to mitochondrial acetyl-CoA, inhibits the decomposition of pyruvate into lactic acid, and regulates metabolic pathways during the activation of dendritic cells. It inhibits the production and maturation of proinflammatory cytokines in dendritic cells induced by lipopolysaccharide (LPS), exerting a therapeutic effect in sepsis (Choi et al., 2020).

Overall, in the TME, LDs in DCs cannot be generally considered proinflammatory or anti-inflammatory because the function of LDs is primarily determined by lipid-associated proteins and internal lipids, which are closely related to the local microenvironment. In addition, the cross-antigen presentation of DCs in the tumor microenvironment is weakened. The relationship between LDs and DCs also plays a key role in the occurrence and progression other diseases, such as obesity, infection, and sepsis.

### Lipid Droplets and T Cells

The cells of the innate and adaptive immune systems, including T cells, have been shown to rely on different aspects of lipid metabolism to develop and function (Howie et al., 2017b). The microenvironment, including cytokines and costimulatory signal molecules, guides T cells to develop along different functional pathways. To a large extent, this is due to the distinct responses of T cell subpopulations to different metabolic stresses. LDs are derived from the endoplasmic reticulum, and DGAT1 and 2 esterify free acylated fatty acids to diacylglycerols to form more inert triacylglycerols, which are stored in the LD core. DGAT1 is responsible for the esterification of exogenous fatty acids into TG, while DGAT2 is considered to have advantages in the esterification of endogenous synthetic FAs (Bhatt-Wessel et al., 2018).

Th1, Th2, and Th17 effector T cells primarily rely on glucose uptake and glycolysis to function and maintain their stability, while regulatory T cells, a class of immunosuppressive Foxp3+ T cells, do not rely on glycolysis, and their mitochondrial oxidative phosphorylation and FAO levels are high (Huynh et al., 2015). Regulatory T cells (Tregs) are a subset of T cells that inhibit the activation and proliferation of other immune cells and play an important role in the suppression of rejection. Acetyl-coenzyme A carboxylase 1 (ACC1) is key for fatty acid generation in T cells. In addition, regardless of whether it is for mice or humans, inhibiting ACC1 not only inhibits fatty acid synthesis but also interferes with glucose metabolism through glycolysis and TCA, which can inhibit proinflammatory Th17 cells and CD8+ T cell induction and proliferation and promote the production of Foxp3 (+) Treg cells with immunosuppressive function, suggesting that endogenous fatty acid synthesis is needed for Th17 cells to develop (Berod et al., 2014). In the absence of exogenous long-chain free fatty acids, the formation of Foxp3+ Treg cells induced by TGFβ is inhibited (Michalek et al., 2011). These results indicate that endogenous fatty acids may be a key player in the development and differentiation of T cells. Regulatory T cells exhibit an enhanced ability to store lipids in LDs and highly express the enzymes necessary for triacylglycerol synthesis [diacylglycerol acyltransferase (DGAT1&2)] (Howie et al., 2019).

The accumulation of acylated long-chain fatty acids in T cells threatens cell survival. In general, there are three non-mutually exclusive intracellular mechanisms for avoiding the toxic effects of acylated long-chain fatty acids: conversion to triglycerides and storage as LDs, increased β-oxidation in the mitochondria, and removal *via* autophagy (Listenberger et al., 2003; Nguyen et al., 2017). In regulatory T cells, the conversion of diacylglycerols to triacylglycerols has three purposes: lipid storage, avoidance of the toxic effects of acylated long-chain fatty acids, and limitation of the signaling activation of protein kinase C by free diacylglycerol. Foxp3 can upregulate the activity of complexes in the electron transport chain, reprogram and regulate Treg metabolism, drive Tregs to adopt the FAO-OXPHOS metabolism model, and enhance resistance to lipotoxicity (Howie et al., 2017a, 2019). mTOR plays a key role in regulating T cell activation, differentiation, and function, and this ability of mTOR is closely related to its role in promoting T cell metabolic reprogramming. As AMPK and mTOR regulate lipid oxidation and enrich Treg differentiation in the body, inhibition of mTOR and activation of AMPK can lead to increased oxidation of exogenous FAs and promote Treg differentiation (Michalek et al., 2011). Therefore, the availability of metabolic substrates is the driving factor of T cell fate. Instead of using extracellular FAs directly, memory T cells use extracellular glucose to support FAO and oxidative phosphorylation (OXPHOS), indicating that lipids must be synthesized to produce the substrate required for FAO. The inherent lipolysis of cells has a decisive effect on the fate of memory T cells. Memory CD8+ T cells lacking LDs still use inherent lipophilic ER-resident triglycerides for maintenance and function (O’Sullivan et al., 2014). In response to low glucose, naïve T cells and central memory T cells increase oxidative phosphorylation, rely on fatty acid metabolism, increase autophagy, and redirect glutamine to induce fatty acid and pyruvate metabolic pathways (Ecker et al., 2018). The increased dependence on fatty acid oxidation and synthesis after T cell activation is associated with decreased IFN-γ expression. Effector memory T cells, however, do not upregulate fatty acid synthesis and can maintain high levels of IFN-γ production at low glucose levels after T cell activation. In general, different types of T cells sense multiple stresses of lipid metabolism, the translation of which may affect cellular differentiation. When autophagy deficiency occurs, transmission electron microscopy shows the accumulation of LDs in IFT20KD cells, suggesting that T cell autophagy requires IFT20 and that the intraflagellar transporter IFT20 controls lysosomal biogenesis by regulating Golgi posttransport of acidic hydrolases (Finetti et al., 2020).

**FIGURE 6:**
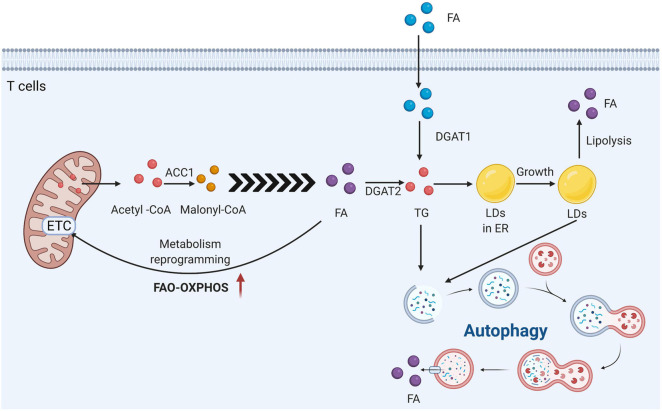
T cells rely on the different aspects of lipid metabolism to develop function and differentiation. Acetyl-Coenzyme A carboxylase 1 (ACC1) is the key for the fatty acid generation in T cells. It can convert Acetyl-CoA into Malonyl-CoA. LDs are derived from the endoplasmic reticulum, and DGAT1 and 2 esterify free acylated fatty acids to diacylglycerols to form more inert triacylglycerols, which are stored in the LDs core. DGAT1 is responsible for the esterification of mainly exogenous fatty acids into TG, while DGAT2 is considered to have advantages in the esterification of endogenous synthetic FA. In general, there are three non-mutually exclusive intracellular mechanisms to avoid the toxic effects of acylated long-chain fatty acids: conversion to triglycerides and storage as LDs, increased β-oxidation in the mitochondria, and removal *via* autophagy.

Overall, T cells rely on different aspects of lipid metabolism to develop, function and differentiate. The heterogeneity of fatty acid metabolic substrates is also closely related to the different functions and phenotypes of T cells (Figure 6). The specific mechanisms regarding their regulation and interactions remain unclear.

## Diseases Related to Lipid Droplets and Immune Cells

### Atherosclerosis

Atherosclerosis is one of the leading causes of morbidity and mortality globally. Atherosclerosis is a chronic and progressive inflammatory disease involving the innate and adaptive immune systems that is characterized by endothelial dysfunction, intimal lipid deposition, smooth muscle cell proliferation, apoptosis, and necrosis (Abdolmaleki et al., 2019; Gąsecka et al., 2021). This silent progressive disease, affecting large and mid-sized arteries, with the absence of a luminal elastin barrier and exposure of a dense collagen/proteoglycan, can block blood flow and cause ischemic impairment of downstream tissues or sudden rupture of atherosclerotic plaques (Wolf and Ley, 2019). It has been reported that accumulated lipids represent a central characteristic of inflammatory atherosclerosis and are related to triggering the immune response (Shah, 2019). Thus, this type of disorder exemplifies the connection between abnormal lipid metabolism and immune disease.

The atherogenic process begins with the accumulation of plasma lipoproteins (e.g., low-density lipoprotein, LDL) in the intima, the innermost layer of the artery wall, which experiences flow perturbation and endothelial dysfunction. Vascular wall cells, including smooth muscle cells and endothelial cells, when stimulated by abnormal lipid deposition, mediate leukocyte recruitment and vascular remodeling, as well as the sustained release of proinflammatory cytokines and chemokines. Monocytes/macrophages are also activated due to inflammatory cytokine storms (Ait-Oufella et al., 2011). Differentiated macrophages internalize native and modified lipoproteins (e.g., oxidation resulting in oxLDL) by expressing scavenger receptors and subsequently converting them into cholesterol-rich foam cells, a hallmark of atherosclerosis, leading to a series of complex inflammatory cascades, including developing atherosclerotic lesions, giving rise to fatty streaks that can shape the architecture of advanced plaques. Eventually, the plaque may rupture, causing thrombus formation and possible subsequent infarction of downstream tissue (Weber et al., 2008; Wolfs et al., 2011). Levels of plasma cholesterol, LDL cholesterol, and apolipoproteins are highly correlated with clinical atherosclerosis. Genetic knockout of LDLR (the LDL receptor) or ApoE (apolipoprotein E) in mice, which elevate plasma cholesterol levels, leads to atherosclerosis in C57BL/6 mice (Ishibashi et al., 1993).

Various molecular steps are involved in initiation of the atherosclerotic process. Activation of endothelial cells induces the expression of leukocyte adhesion molecules, such as endothelial-selectin (E-selectin), intercellular adhesion molecule-1 (ICAM-1), vascular cell adhesion molecule-1 (VCAM-1), and P-selectin. Migration and infiltration of monocytes are stimulated by the subsequent production of monocyte chemoattractant protein-1 (MCP-1) (Clinton et al., 1992; Gimbrone and García-Cardeña, 2016). Subsequently, monocytes differentiate into macrophages in response to the action of macrophage colony-stimulating factor (M-CSF) (Jones and Ricardo, 2013).

Lipoproteins are modified by oxidizing agents, proteases, and lipases, generating oxLDLs, acetylated LDLs, etc., (Oörni et al., 2000). Macrophages take up oxidized low-density lipoproteins (oxLDL) *via* diverse mechanisms, such as the action of scavenger receptors such as CD36, cholesterol hydrolysis, or phagocytosis, converting to foam cells filled with cytoplasmic LDs, and lipids can be effluxed *via* lipolysis or lipophagy (Chang et al., 1997; Lee and Choi, 2020). C. pneumonia infection results in the chronic inflammatory disease atherosclerosis, leading to lipid droplet-containing foam cell formation within arteries of the host (Libbing et al., 2019). A previous study demonstrated that LDs can be enriched with free cholesterol from hyperlipidaemic serum due to hydrolysis and rearrangement of cellular cholesterol in foam cells (Mori et al., 2001).

The classical categorization of macrophages includes two phenotypes, M1 and M2. Monocytes differentiate into M1 or M2 macrophages in response to exposure to GM-CSF or M-CSF, respectively (Shapouri-Moghaddam et al., 2018). Both types of macrophages are found in atherosclerotic lesions. M1 macrophages are regarded as proinflammatory cells since they secrete various proinflammatory factors, such as TNF-α, IL-1α, and IL-1β, which can recruit inflammatory cells and accelerate plaque development and necrotic core formation, leading to thrombotic events (Chistiakov et al., 2015a). In contrast, M2 (or alternatively polarized) macrophages play an anti-inflammatory and atheroprotective role by restraining cell recruitment and tissue remodeling. They can also reduce the formation of foam cells and strengthen plaque stability. Moreover, M2 macrophages are further subclassified into four subsets (M2a, M2b, M2c, and M2d), depending on their mechanism of activation (Koelwyn et al., 2018).

Both innate immunity and adaptive immunity are crucial for the advancement and expansion of atherosclerosis. The innate response begins with the stimulation of monocytes/macrophages in the vessel walls and is followed by multiple adaptive responses that are modulated by T and B cells. Some effector cells, such as mast cells and eosinophils, may also contribute to atherosclerotic disease (Bot et al., 2007). It has been determined that cell-derived IFN-γ and IL-6 play a proinflammatory role in lesion development. Moreover, eosinophils and activated IgE can promote atherosclerosis progression (Xin et al., 2020). From a disease treatment perspective, immune modulation of T and B cell-mediated responses represents an attractive antiatherosclerotic therapeutic strategy.

### Mycobacterium tuberculosis

*Mycobacterium tuberculosis* (M.tb), the causal agent of tuberculosis and the leading cause of death among individuals living with HIV, is likely the most successful human pathogen. The interplay between M.tb and lipid metabolism and the host immune response creates a complex pathogen-host interaction (Bailo et al., 2015). In particular, lipids serve as the key mediators of this interaction, not only as nutrient sources for M.tb but also as modulators of host immune responses. Interestingly, this bacterium seems to preferentially utilize host lipids to maintain self-metabolic activity (Pandey and Sassetti, 2008). Moreover, there are specific host pathways, such as the PPARγ and LXR transcriptional regulators, that have revealed the mechanisms by which host immunity alters the bacterial microenvironment. One hallmark of tuberculosis infection is the accumulation of M.tb-infected foamy macrophages containing large lipid bodies (Wong et al., 2020). In this review, we focus on emerging concepts and the current understanding of lipid utilization in M.tb, how the immune response modulates lipid homeostasis in both the bacterium and the host, and how these pathways are likely to be manipulated therapeutically.

As a highly successful intracellular pathogen, M.tb can tolerate harsh conditions and reside long-term within its host by refining its metabolism. Studies conducted over the past 60 years have clearly shown that M.tb relies on fatty acids and cholesterol as important nutrients during infection (Bloch and Segal, 1956; Chang and Guan, 2021). There are two types of growth behavior in bacteria: diauxic and coutilization of carbon sources. M.tb has a preference for the latter, and it can catabolize multiple carbon sources simultaneously through compartmentalization of separate metabolic processes (de Carvalho et al., 2010).

Fatty acids rather than carbohydrates are the primary carbon source for M.tb during infection. M.tb utilizes fatty acids for specific purposes, including as substrates for beta-oxidation, acyl primers for polyketide lipid synthesis, or by incorporating them into phospholipids and/or triacylglycerol (TAG) (Lovewell et al., 2016). Most importantly, M.tb β-oxidizes fatty acids to produce energy in its crucial central metabolism. Second, fatty acids are also donated to the production of virulence-associated molecules, such as polyketide lipids phthiocerol-dimycocerosate (PDIM), polyacylated trehalose (PAT), sulfolipid (SL), and mycolic acids. Third, fatty acids can be assimilated directly into cell membrane phospholipids, which maintain cytoplasmic membrane integrity or are converted into cellular TAG, which functions as a carbon storage molecule (Trivedi et al., 2004; Daniel et al., 2011; Baran et al., 2020).

Several lines of evidence indicate that lipid metabolic pathways not only serve as a source of carbon and energy but can also protect M.tb from stress. For example, TAG synthesis reduces the M.tb growth rate and antibiotic sensitivity by reducing acetyl-CoA availability. Cytosolic redox can be influenced through the balance of fatty acid catabolism and anabolism (Jansen et al., 2020). Host lipids have been shown to play a dominant role in optimal growth and persistence of M.tb during infection. One possibility for the mechanisms of acquiring host lipids is that fatty acids from LDs or lipid bilayers originate from the host cell for use by M.tb. Another possibility is that M.tb acquires cholesterol and fatty acids directly from lipoproteins within the macrophage endocytic network trafficking in a soluble form (Bonds et al., 2020).

During tuberculosis infection, the immune response of the host can cause a variety of changes in both intracellular and extracellular bacteria. Coordinated efforts of the innate and adaptive immune systems are required (Crowther and Qualls, 2020). Inflammatory signals triggered by many microbial pathogens, such as M.tb and Chlamydia pneumoniae, induce the accumulation of LDs in immune cells, such as neutrophils, eosinophils, and macrophages. Macrophages then adopt a foamy morphology and thus are termed foam cells. Interestingly, in foam macrophages, mycobacteria are present with phagosomes that are very close to LDs and transition to a dormant state. Moreover, the accumulation of lipids by M.tb within foam macrophages is primarily a result of the incorporation of fatty acids derived from host TAG in a process largely mediated by mycobacterial triacylglycerol synthase 1 (Maphasa et al., 2020).

Several clarified mechanisms underlie immune-mediated alterations in macrophage lipid homeostasis. One major mechanism behind this modulation involves the infection-dependent activation of peroxisome proliferator-activated receptors (PPARs). M.tb infection can increase PPAR-γ expression in infected cells in a Toll-like receptor 2 (TLR2)-dependent manner, and activation of these signaling pathways leads to lipid droplet accumulation. Furthermore, host-cell lipid signaling programs such as the CD36 pathways are now being revealed as important routes for determining outcomes during M.tb infection. Finally, lipid accumulation in the M.tb-infected host could exert additional effects on the pathogen. It not only provides a growth substrate for the bacterium and promotes a hyperinflammatory environment but also increases the intracellular lipid pool, which can trigger an anti-starvation response and inhibit autophagy and lysosome acidification (Parihar et al., 2014; Paik and Jo, 2020; Chen et al., 2021).

### Cancer

Although prominently found in adipose tissue, LDs can exist in all cell types and tissues (Lynch and Scott, 1951). Higher LD contents have been reported in cancer cells and cancerous tissues such as colorectal cancer, breast and prostate cancers, hepatocellular carcinoma, renal cell carcinoma and glioblastoma, and LDs can extensively mediate proliferation, invasion, metastasis and chemotherapy resistance in various cancers. In this article, we summarized the indispensable role that LDs play in carcinogenesis, malignant development of cancers, and immune cells.

Lipid metabolic enzymes are responsible for the synthesis or degradation of LD components, such as LPCAT2, cytosolic phospholipase 2 (cPLA2), sterol *O*-acyltransferase 1 (SOAT1), etc. Sterol regulatory element-binding protein-1 (SREBP-1) is a key transcription factor in regulating lipid synthesis and uptake. Recent studies have revealed that SREBPs are highly activated in various cancers and promote tumor growth, which shows promise for potential drugs targeting SREBP-1-driven lipid metabolism as anticancer agents (Brown and Goldstein, 1997). In addition, LD coat proteins (Perilipins) and fatty acid-binding proteins (FABPs) are also involved in the regulation of LD formation and trafficking in cancer cells. A-FABP expression in macrophages promotes breast cancer growth and metastasis through IL-6/STAT3 signaling, whereas E-FABP expression in macrophages strengthens type I interferon β (IFN β) responses against tumor progression (Zhang et al., 2014; Hao et al., 2018).

Lipid droplets are composed of a monolayer of phospholipids as well as a hydrophobic core of neutral lipids (consisting of primarily triacylglycerols and cholesterol esters). Increased storage of lipids in LDs is beneficial for the malignant development of cancers. This was illustrated by a study in a breast cancer cell line, where LD abundance was shown to correlate with the degree of invasiveness from non-malignant MCF10A cells to highly malignant MDA-MB-231 cells (Nieva et al., 2012). Intracellular excess fatty acids and cholesterol stored in LDs prevent lipotoxicity and endoplasmic reticulum stress. Moreover, increased LD contents could expand the source of lipid substrates and energy to support the metabolic requirement of proliferative cancer cells. For example, in the tumor microenvironment (TME), LDs could provide energy for aggressive cancer to trigger metastatic cloning. The lipid-rich tumor microenvironment also resulted in defective antitumor properties in dendritic cells (DCs). DCs, a type of professional antigen-presenting cell, can initiate and sustain T cell-dependent anticancer immunity. A previous study found that DCs with high LD content were unable to present tumor-associated antigens or stimulate allogeneic T cells in tumor-bearing mice. The accumulation of LDs was caused by increased uptake of extracellular lipids (Veglia et al., 2017). Another study verified that the abnormal build-up of LDs in tumor-associated DCs could be activated by the intrinsic ER stress-dependent XBP1 pathway, which promoted ovarian cancer progression (Cubillos-Ruiz et al., 2015). The effects of lipid regulation on DC function in cancer were studied by using 5-(tetradecyloxy)-2-furoic acid (TOFA) to suppress lipid accumulation, which led to restoration of the functional capacity of DCs and enhanced the activity of the antitumor T-cell response (Herber et al., 2010). The molecular mechanisms underlying defective antigen presentation by high LD content remain to be elucidated. LD accumulation in cancer cells may modulate the effectiveness of antitumor immune responses and therefore provide a promising therapeutic target.

## Conclusion

In recent years, LDs have received worldwide attention, and breakthrough results have been achieved in many fields, especially with respect to the contribution of LDs to the immune system. With the popularity and gradual development of the concept of metabolic immunity in recent years and the characteristics of LDs as a key intermediate bridge connecting metabolism and immunity, the role and specific mechanism of LDs in immune and metabolic diseases have been revealed by an increasing number of studies, which have greatly improved and supplemented understanding of the functional and structural characteristics of LDs. Although many studies have been conducted on the biogenesis of LDs, lipid degradation, and the physical and functional connections between LDs and other organelles in the body, many mechanisms and details are still unknown and need to be further explored. Lipid drops are closely related to the immune system, especially the synthesis and secretion of some cytokines of lipid bioactivity, which are regulated by lipid drops. Previously, it was thought that LDs support infection by providing substrates to synthesize membrane and produce energy, ensuring the stability of the pathogen membrane structure and the persistent development of infection. However, recent research results seem contradictory. They assert that in response to infection, mammalian fat droplets assemble antibiotics and immune proteins to form a complex that fights pathogen invasion. Thus, the anti-infection effect of LDs on the immune system remains controversial. One possible explanation for this controversy is that the function of LDs is variable and may be related to the organism, tissue, cell, subcellular location, etc. Similar to the functional and morphological heterogeneity of mitochondria in different microenvironments, as a kind of organelle, whether the heterogeneity of LDs is not limited to their number, size, and composition and whether their function dynamically displays different characteristics, depending on the microenvironment in which they are located remain to be further explored. Although the strong correlation between LDs and the immune system plays an encouraging role in atherosclerosis and anti-TB bacilli, a serious reality is that there is still a lack of safe and effective therapeutic targets that can be applied in clinical practice at present. This partly explains why the connection between them still has much uncertainty. Therefore, a thorough understanding of the role of LDs in the immune system and the mechanism of regulating immune cells is of great significance for the development of new anti-infection drugs and the improvement of therapeutic targets for atherosclerosis.

## Author Contributions

MZ and LX: conceptualization. WZ: writing—original draft preparation. WZ, LZ, and LX: writing—review and editing. WZ and SY: visualization. MZ: supervision. All authors have read and agreed to the published version of the manuscript.

## Conflict of Interest

The authors declare that the research was conducted in the absence of any commercial or financial relationships that could be construed as a potential conflict of interest.

## Publisher’s Note

All claims expressed in this article are solely those of the authors and do not necessarily represent those of their affiliated organizations, or those of the publisher, the editors and the reviewers. Any product that may be evaluated in this article, or claim that may be made by its manufacturer, is not guaranteed or endorsed by the publisher.
